# Construction of the survival nomograms for colon cancer patients of different ages based on the SEER database

**DOI:** 10.1007/s00432-023-05323-8

**Published:** 2023-08-28

**Authors:** Yuzhou Yang, Peng Xu, Cheng Zhang

**Affiliations:** 1 Department of General Surgery, General Hospital of Northern Theater Command (General Hospital of Shenyang Military Command), Shenyang, Liaoning Province China; 2 Jinzhou Medical University, Jinzhou, Liaoning Province China

**Keywords:** Colon cancer, SEER, Nomogram, Early-onset colon cancer, Late-onset colon cancer

## Abstract

**Introduction:**

Three nomograms for predicting the outcomes of early- and late-onset colon cancer (COCA) among patients not stratified by age were constructed using data in the Epidemiology and End Results (SEER) database (1975–2019). The accuracy of the nomogram was then assessed.

**Method:**

Clinical data of 6107 patients with COCA were obtained from the SEER database. The patients were randomly divided into training and validation cohorts in a ratio of 7:3. Univariate and multivariate COX analyses of factors that could independently impact the prognosis of COCA were performed, and the corresponding nomograms for early-onset and late-onset COCA were constructed. Calibration curves, ROC curves, and C-index were used to determine the predictive accuracy. The discriminatory ability of the nomograms to assess their clinical utility, which was compared with the TNM staging system of the 8th edition of AJCC, was verified using survival analysis.

**Result:**

Tumor primary site, ethnicity, and serum carcinoembryonic antigen (CEA) level significantly impacted the prognosis of colon cancer. Race, brain metastasis, and CEA were independent factors for predicting COCA prognosis. C-index, ROC, and calibration curves demonstrated that the three nomograms were accurate and superior to the traditional TNM staging system. Among the three nomograms, the early-onset COCA nomogram had the highest predictive accuracy, followed by that of colon cancer not stratified by age.

**Conclusion:**

Three nomograms for patients not stratified by age, early-onset colon cancer, and late-onset colon cancer were constructed. The accuracies of the nomograms were good and were all superior to the conventional TNM staging system. The early- and late-onset COCA nomograms are useful for clinical management and individualized treatment of COCA patients at different ages.

## Introduction

Colorectal cancer (CRC) is the fourth most fatal cancer globally, killing nearly 9 million people annually. In addition to an aging population and dietary habits, several factors, such as obesity, lack of physical activity, and smoking, increase the risk of CRC (Dekker et al. 2019). The USA had 147,950 new cases and 53,200 deaths in 2020 (Siegel et al. 2020). Patients with colon cancer (COCA) mainly present with small cell anemia, rectal bleeding, chronic abdominal pain, and changes in bowel habits (Benson et al. 2018). The median age of COCA onset is 67 years. Although only 12% of COCA patients are aged under 50 years, the incidence of CRC in individuals younger than 50 has increased by approximately 2% per year in the USA since the 1990s (Siegel et al. 2020).

Early-onset COCA refers to COCA diagnosed at < 50 years of age. The incidence of early-onset COCA is increasing worldwide, and its pathogenesis is still unclear. Early-onset COCA has different clinical, pathological, and molecular features. Compared with late-onset COCA, which refers to COCA diagnosed at age > 50 years, early-onset COCA mostly occurs in the descending colon, is mostly diagnosed in the late stage, and is poorly differentiated. The risk factors of early-onset COCA are not the same as those of late-onset COCA (Zaborowski et al. 2021). Comparative analysis of gene expression in early-onset and late-onset COCA identified 88 genes specific to early-onset COCA (Jandova et al. 2016). CLC and IFNAR1 differ in somatic gene expression between younger and older COCA patients, highlighting the genomic complexity of COCA in patients of different ages (Ågesen et al. 2011).

The tumor-node-metastasis (TNM) staging system, proposed by the International Union Against Cancer (UICC) and the American Joint Committee on Cancer (AJCC), is the standard method for staging malignant tumors and is widely used to assess cancer prognosis (Hari et al. 2013). The TNM staging system plays an important role in formulating treatment strategies, assessing treatment outcomes, predicting the survival time of patients after surgery, and the management of COCA patients (Delattre et al. 2022). In addition, studies have shown that other factors such as age, serum carcinoembryonic antigen (CEA) level, race, and tumor site are also strongly associated with tumor development in individual cases (Wang et al. 2015; Liang et al. 2018; Biller and Schrag 2021). Clinically, COCA can easily metastasize to other parts of the body, with the lung and liver being the most common metastatic organs, followed by bone and brain (Wang et al. 2020). The TNM staging system has two main limitations; it assesses the risk of individual patients by incorporating only three variables (T-stage, N-stage, and M-stage) and other risk factors such as age, gender, race, and tumor size, among others, cannot be incoperated (Duijster et al. 2021).

Nomograms have been applied in clinical studies related to prognosis and have gained wide acceptance (Song et al. 2018a; He et al. 2018). However, studies on the differences in the predictive accuracy of nomograms for COCA patients at different ages remain scanty. Herein, we analyzed a large amount of colon cancer data from the Surveillance, Epidemiology and End Results (SEER) program (1975–2019) and constructed three nomograms: not stratified-by-age nomograms, early-onset colon cancer (COCA) nomogram, and late-onset COCA nomogram to predict individualized survival time of colon cancer patients at any age. The accuracy of each nomogram was assessed.

## Materials and methods

### Patients selection

**Fig. 1:**
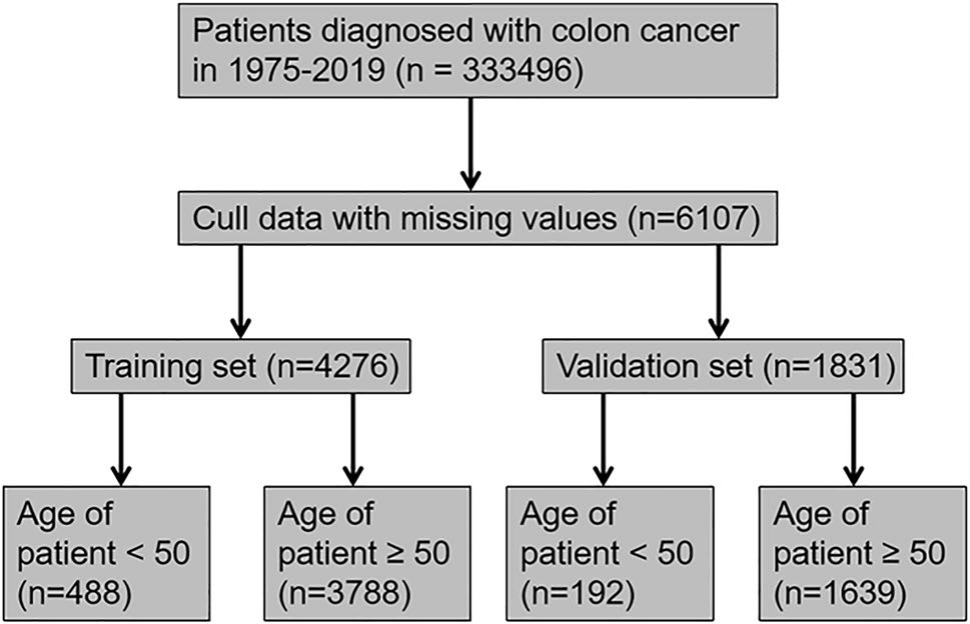
Flow chart for selecting COCA patients included in the present study

This was a retrospective cohort study in which data were obtained from the surveillance, epidemiology, and final results database (www.seer.cancer.gov). The SEER database, updated every year since 1973, comprises the incidence, prevalence, and mortality data of patients with various tumors and can be used to analyze tumor trends. The data for patients diagnosed with colon cancer between 1975 and 2019 were retrieved using SEER * Stat software version 8.4.0.1. The database contained data for 333,496 COCA patients. Patients with invalid or missing data were excluded from the study. Patients were randomly divided into training and validation cohorts in a ratio of 7:3, and patients in each group were divided into two groups based on age (> 50 and < 50). The procedure for data retrieval is shown in Fig. 1.

### Clinical variables and outcomes

The clinical variables analyzed in this study include age, race, gender, tumor primary site, tumor size, TNM stage based on the 8th edition of AJCC guidelines, serum CEA level, nerve invasion, lymph node metastasis, liver metastasis, lung metastasis, bone metastasis, and brain metastasis. The primary outcome analyzed was the overall survival (OS), which refers to the time from diagnosis to death from any cause or the end of the follow-up period.

### Univariate and multivariate COX regression analyses

The 6107 MCC patients included in this study were randomly divided into two groups in the ratio of 7:3 using R software (version 4.1.3). Data for patients in the training set were used to build the nomograms (*n* = 4276), while that of those in the validation set were used to verify the predictive accuracy of the nomograms (*n* = 1831). Univariate Cox proportional risk regression analysis was performed to evaluate the contribution of each clinical variable in predicting COCA prognosis. These variables included tumor characteristics (T stage, N stage, M stage, tumor size, primary tumor location, other organ metastases, etc.), demographic variables (race, gender, and age), and serum carcinoembryonic antigen level. Demographic variables, TNM stage, and statistically significant factors were then included in the multivariate COX regression analysis to calculate hazard ratios (HRs) at 95% confidence intervals (CIs). AJCC staging was excluded from the Cox regression because combining T, N, and M stages could interfere with AJCC staging.

### Construction of prognostic nomogram

Demographic variables (race, gender, age) and indicators with *p* less than 0.05 in the univariate and multivariate analyses were included in constructing a predictive nomogram, and the no-model plots were constructed using “Survival,” “Foreign,” and “RMS” packages in the R software. RMS program package is to construct the nomogram. Each variable in the nomogram was assigned a score, and the final multiple scores were summed to give an overall score for predicting OS at months 6 and 18, and the performance of the nomogram was measured by the consistency index (C-index). The median risk score was used as the cutoff value to divide the patients into high-risk and low-risk groups. Kaplan**–**Meier (KM) survival curves were used to fit the correlation between survival time and predicted scores in the high-risk and low-risk groups. Models were also constructed based on the TNM scoring system of the 8th AJCC edition. The C-index was calculated, and the ROC, correction, and survival curves for early-onset and late-onset colon cancer were plotted. The reliability and applicability of the models were compared.

## Result

### Patient characteristics

A total of 6107 COCA patients were obtained from the SEER database. Of these, 4276 were classified into the training cohort and 1831 in the validation group. Of the patients in the training cohort, 488 patients (11.4%) in the training cohort were < 50 years old, the majority (75.9%) were white, while the rest were black or other races, and 2154 (50.3%) were males. For the validation cohort, 916 patients (50%) were males, and 192 patients (48.0%) were < 50 years of age (Table 1**)**.

**Table 1:** The demographics and pathological characteristics of patients included in the present study

Characteristic	Train cohort (*n* = 4276)	Test cohort (*n* = 1831)
Age		
< 30	24 (0.5%)	6 (0.3%)
30–44	249 (5.8%)	95 (5.1%)
45–59	1003 (23.4%)	432 (23.5%)
60–74	1563 (36.5%)	685 (37.4%)
≥ 75	1437 (33.6%)	613 (33.4%)
Race		
Black	422 (9.8%)	190 (10.3%)
White	3248 (75.9%)	1375 (75%)
Other	606 (14.1%)	266 (14.5%)
Sex		
Male	2122 (49.6%)	915 (49.9%)
Female	2154 (50.3%)	916 (50%)
Primary site		
Cecum (180)	1005 (23.5%)	405 (22.1%)
Colon ascendens (182)	994 (23.2%)	413 (22.5%)
Flexura hepatica coli (183)	233 (5.5%)	94 (5.1%)
Colon transversum (184)	450 (10.5%)	205 (11.1%)
Splenic flexure of colon (185)	145 (3.3%)	72 (3.9%)
Colon descendens (186)	250 (5.8%)	106 (5.7%)
Colon sigmoideum (187)	1115 (26.0%)	496 (27%)
Cross-overlap span (188)	67 (1.5%)	29 (1.5%)
Other (189)	17 (0.3%)	11 (0.6%)
T stage		
Tis	40 (0.9%)	10 (0.5%)
T0	3 (< 0.1%)	0
T1	474 (11.0%)	218 (11.9%)
T2	548 (12.8%)	265 (14.4%)
T3	2261 (52.8%)	963 (52.5%)
T4a	661 (15.4%)	254 (13.8%)
T4b	289 (6.7%)	121 (6.6%)
N stage		
N0	2353 (55.0%)	1046 (57.1%)
N1a	502 (11.7%)	228 (12.4%)
N1b	584 (13.6%)	239 (13%)
N1c	141 (3.2%)	51 (2.7%)
N2a	353 (8.2%)	135 (7.3%)
N2b	343 (8%)	132 (7.2%)
M Stage		
M0	3708 (86.7%)	1590 (86.8%)
M1a	309 (7.2%)	145 (7.9%)
M1b	128 (2.9%)	47 (2.5%)
M1c	131 (3%)	49 (2.6%)
CEA		
Negative	2538 (59.3%)	1065 (58.1%)
Positive	1738 (40.6%)	766 (41.8%)
Perineural invasion		
No	3644 (85.2%)	1550 (84.6%)
Yes	632 (14.7%)	281 (15.3%)
Liver metastasis		
No	3902 (91.2%)	1664 (90.8%)
Yes	374 (8.7%)	167 (9.1%)
Lung metastasis		
No	4175 (97.6%)	1796 (98%)
Yes	101 (2.3%)	35 (1.9%)
Bone metastasis		
No	4262 (99.6%)	1824 (99.6%)
Yes	14 (0.3%)	7 (0.3%)
Brain metastasis		
No	4271 (99.8%)	1829 (99.8%)
Yes	5 (0.1%)	2 (0.1%)
LN metastasis		
No	4203 (98.2%)	1800 (98.3%)
Yes	73 (1.7%)	31 (1.6%)
Tumor size		
< 5 cm	2264 (52.9%)	989 (54%)
5–10 cm	1654 (38.6%)	695 (37.9%)
≥ 10 cm	358 (8.3%)	147 (8%)
Status		
Alive	3850 (90%)	1667 (91%)
Dead	426 (10%)	164 (8.9%)

### Univariate and multivariate COX regression analysis

Firstly, one-way COX regression analysis for age, race, gender, tumor primary site, tumor size, TNM stage of the 8th edition of AJCC, serum carcinoembryonic antigen, nerve invasion, lymph node metastasis, liver metastasis, lung metastasis, bone metastasis, and brain metastasis were performed. Several factors, including primary tumor site, tumor size, ethnicity, serum carcinoembryonic antigen, nerve invasion, lymph node metastasis, liver metastasis, lung metastasis, bone metastasis, and brain metastasis, were significantly associated with COCA prognosis (*p* < 0.05). Primary tumor site, ethnicity, brain metastasis, serum carcinoembryonic antigen, and nerve invasion were independent factors associated with COCA prognosis. The univariate and multivariate COX regression analysis results are shown in Table 2.

**Table 2:** Univariate and multivariate analyses of factors linked to COCA prognosis

Characteristic	Univariate analysis		Multivariate analysis	
	Hazard rate (95%CI)	*p* value	Hazard rate (95%CI)	*p* value
Age				
< 30	1			
30–44	0.72 (0.09–5.77)	0.758		
45–59	0.99 (0.14–7.20)	0.994		
60–74	1.80 (0.25–12.88)	0.559		
≥ 75	3.98 (0.56–28.36)	0.168		
Race				
Black	1		1	
White	0.97 (0.71–1.32)	0.864	1.06 (0.77–1.46)	0.708
Other	0.58 (0.38–0.89)	0.012*	0.64 (0.42–1.00)	0.046*
Sex				
Male	1			
Female	1.00 (0.83–1.21)	0.982		
Primary site				
Cecum (180)	1		1	
Colon ascendens (182)	0.86 (0.66–1.12)	0.258	1.14 (0.87–1.49)	0.335
Flexura hepatica coli (183)	0.63 (0.39–1.02)	0.059	0.78 (0.48–1.28)	0.328
Colon transversum (184)	0.96 (0.69–1.33)	0.818	1.11 (0.79–1.54)	0.551
Splenic flexure of colon (185)	0.68 (0.37–1.22)	0.197	0.76 (0.42–1.38)	0.361
Colon descendens (186)	0.80 (0.52–1.25)	0.382	0.99 (0.63–1.54)	0.947
Colon sigmoideum (187)	0.55 (0.42–0.74)	< 0.001***	0.64 (0.48–0.86)	0.002***
Cross-overlap span (188)	1.23 (0.64–2.34)	0.531	1.12 (0.57–2.20)	0.736
Other (189)	2.17 (0.80–5.87)	0.128	2.52 (0.84–7.58)	0.101
T stage				
Tis	1		1	
T0	9.20 (0.58–147.15)	0.117	1.40 (0.07–26.45)	0.823
T1	2.48 (0.34–18.21)	0.371	2.72 (0.37–20.00)	0.325
T2	1.88 (0.26–13.82)	0.535	1.89 (0.26–13.95)	0.530
T3	3.42 (0.48–24.38)	0.220	2.43 (0.34–17.44)	0.377
T4a	7.21 (1.01–51.67)	0.049*	3.55 (0.49–25.72)	0.210
T4b	8.51 (1.18–61.44)	0.034*	3.39 (0.46–24.80)	0.229
N stage				
N0	1		1	
N1a	1.40 (1.01–1.93)	0.041*	1.03 (0.74–1.45)	0.854
N1b	1.40 (1.02–1.89)	0.032	0.87 (0.62–1.21)	0.423
N1c	1.82 (1.07–3.09)	0.027	1.08 (0.62–1.87)	0.782
N2a	2.01 (1.46–2.77)	< 0.001***	1.05 (0.74–1.49)	0.788
N2b	4.36 (3.38–5.62)	< 0.001***	1.63 (1.19–2.23)	0.003**
M stage				
M0	1		1	
M1a	3.54 (2.73–4.60)	< 0.001***	2.02 (1.33–3.06)	< 0.001***
M1b	4.97 (3.58–6.91)	< 0.001***	2.52 (1.56–4.08)	< 0.001***
M1c	6.96 (5.14–9.43)	< 0.001***	3.19 (2.12–4.81)	< 0.001***
CEA				
Negative	1		1	
Positive	2.71 (2.22–3.30)	< 0.001***	1.78 (1.43–2.21)	< 0.001***
Perineural invasion				
No	1		1	
Yes	2.07 (1.66–2.57)	< 0.001***	1.19 (0.94–1.52)	0.155
Liver metastasis				
No	1		1	
Yes	3.74 (3.00–4.66)	< 0.001***	1.23 (0.86–1.76)	0.256
Lung metastasis				
No	1		1	
Yes	2.92 (1.95–4.38)	< 0.001***	0.83 (0.53–1.31)	0.432
Bone metastasis				
No	1		1	
Yes	7.04 (3.49–14.17)	< 0.001***	1.98 (0.94–4.19)	0.075
Brain metastasis				
No	1		1	
Yes	8.98 (2.88–27.96)	< 0.001***	4.24 (1.27–14.22)	0.019*
LN metastasis				
No	1		1	
Yes	5.59 (2.34–5.51)	< 0.001***	0.88 (0.55–1.40)	0.581
Tumor size				
< 5 cm	1		1	
5–10 cm	1.53 (1.25–1.87)	< 0.001***	1.11 (0.90–1.38)	0.332
≥ 10cm	1.72 (1.26–2.36)	< 0.001***	1.04 (0.75–1.45)	0.821

*p < 0.05; **p < 0.01; ***p < 0.001

### Prognostic nomogram construction

**Fig. 2:**
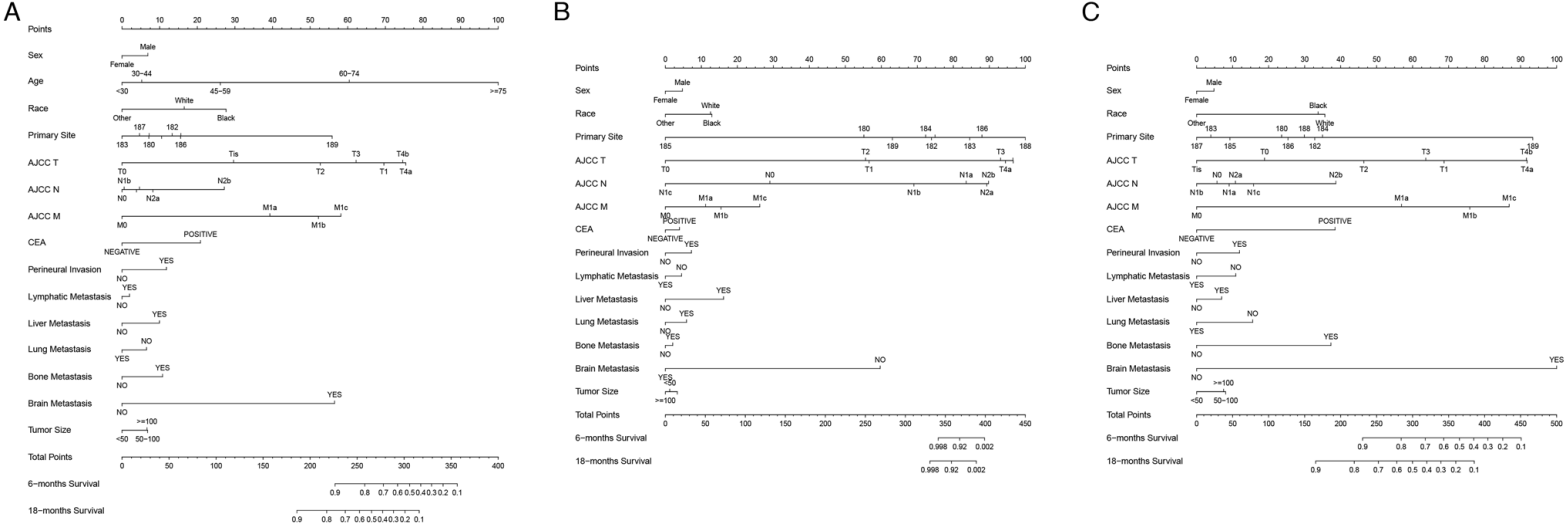
Nomograms for predicting the OS of COCA patients. **A** Nomogram for predicting the prognosis of non-age-stratified COCA patients; **B** nomogram for predicting the prognosis of early-onset COCA cancer; **C** nomograms for predicting the prognosis of late-onset COCA

Nomograms for predicting 6 and 18 months of OS of COCA were constructed using data for patients in the training set (Fig. 2). The risk factors included in the not stratified by age nomogram contained significant demographic variables (race, sex, and age; *p* < 0.05) obtained by univariate and multivariate analyses. The nomogram for predicting the early-onset COCA and late-onset COCA comprised all of the above indicators except age. The nomograms contained scores for each risk factor and the total scores. The nomogram could generally predict the 6-month and 18-month OS of COCA patients (Fig. 2).

### Nomogram calibration and validation

**Fig. 3:**
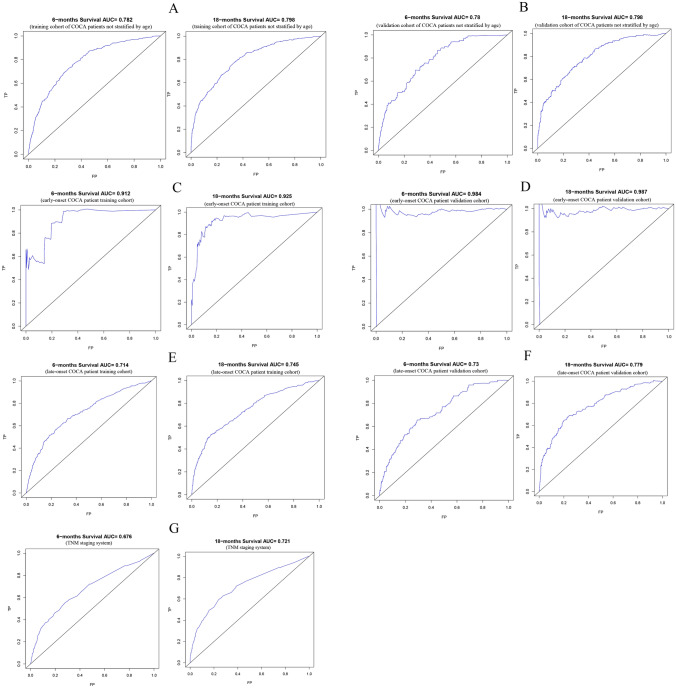
ROC for the 6- and 18-month prognosis prediction of the nomograms and the TNM staging system. **A** ROC for the 6- and 18-month OS prediction of the models for the training cohort of COCA patients not stratified by age; **B** ROC curves for the 6- and 18-months OS prediction of the models for the validation cohort of COCA patients not stratified by age; **C** ROC for the 6 and 18 months’ of OS prediction of the models for the early-onset COCA patients in the training cohort; **D** ROC for the 6- and 18-months OS prediction of the models for the early-onset COCA patients in the validation cohort; **E** ROC for the 6 and 18 months’ of OS prediction of the models for the late-onset COCA patient in the training set; **F** ROC for the 6 and 18 months’ of OS prediction of the models for the late-onset COCA patient in the validation set; **G** ROC for 6 and 18 months for the OS prediction of the TNM staging system

**Fig. 4:**
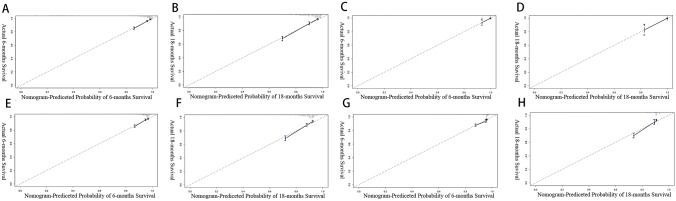
Calibration curves of the nomogram and TNM staging system. **A** Six-month calibration curves for the nomogram not stratified by age; **B** the 18-month calibration curves for nomogram not stratified by age; **C** the 6-month calibration curve for early-onset COCA's nomogram; **D** the 18-month calibration curve for early-onset COCA's nomogram; **E** the 6-month calibration curve for late-onset COCA’s nomogram; **F** the 18-month calibration curve for late-onset COCA’s nomogram; **G** the 6-month calibration curve for TNM staging system; **H** the 18-month calibration curve for TNM staging system

The C-index was used to compare the performance of the nomograms. Particularly, the C-index of the training cohort without stratification by age and the validation cohort was 0.79 (95% CI 0.77–0.81) and 0.81 (95% CI 0.77–0.84), respectively. In the early-onset COCA training set cohort, validation cohort C-index was 0.95 (95% CI 0.91–0.99), 1. In the late-onset COCA training cohort, the validation cohort C-index was 0.72 (95% CI 0.69–0.75), 0.75 (95% CI 0.71–0.79), respectively. The C-index of the nomogram for predicting the OS of patients grouped by the TNM staging based on the 8th edition of AJCC guidelines was 0.68 (95% CI 0.65–0.71). ROC curves were plotted to assess the performance of the models (Fig. 3), and the area under the ROCs of the three for predicting the 6- and 18-month prognosis of COCA patients was greater than 0.7, which was higher than that of the TNM staging AJCC system. In addition, calibration plots of the three prediction models and the TNM staging system for the training cohort were plotted (Fig. 4). We found a high consistency between the predicted survival probabilities and the observed outcomes. The above results demonstrate that the better performance of our models is superior to that of the TNM staging system.

### Survival analyses

**Fig. 5:**
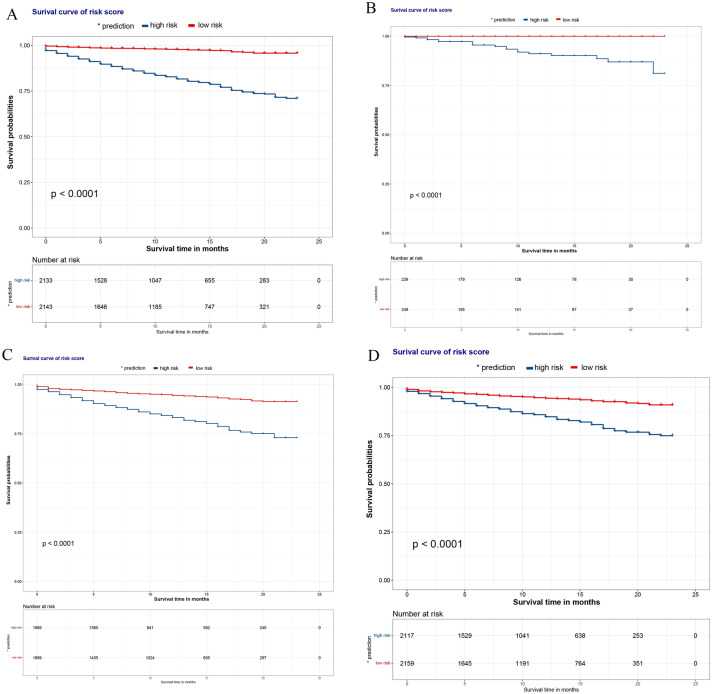
The KM curves for OS prediction of the TNM staging system. **A** KM curves of the models for patients not stratified by age; **B** KM curve of early-onset COCA model; **C** KM curve of late-onset COCA model; **D** KM curve of TNM staging system

The overall risk scores (with the median score as the critical value) of each prognostic factor included in the three nomograms were calculated, and patients were divided into low-risk and high-risk groups. The KM curves were plotted (Fig. 5A–C), which suggested that the prognosis of the low-risk group individuals was better than that of the high-risk group individuals. We also plotted KM curves for the conventional TNM staging system, which still revealed a better prognosis for the low-risk group (Fig. 5D).

## Discussion

COCA is the fifth leading cause of cancer-related deaths worldwide (Sung et al. 2020). A total of 13 factors, including age, gender, and metastatic status, have been associated with COCA (Ge et al. 2019). COCA is prone to distant metastasis, and the site of metastasis is important to COCA prognosis. COCA is highly metastatic to the liver, lungs, brain, and bone, and the risk of death is higher in patients with these metastases than those without (Wang et al. 2020; Chang et al. 2017; Nakamura et al. 2021). In this study, we constructed a non-age stratification, early-onset COCA, and late-onset COCA nomograms using data in the SEER database. The nomograms were validated to have had good discriminatory ability, accuracy, and positive predictive power. The clinical significance of indicators such as race, distant metastasis, and CEA in COCA patients was investigated.

The dataset not stratified by age in this study is one in which the study cohort was not grouped based on age. Many previous studies have demonstrated that for cancer patients, older adults have a shorter OS survival than their younger opposites (Tai et al. 2022; Chen et al. 2020; Badic et al. 2021; Pilleron et al. 2021). In addition, Kuai et al. constructed a nomogram of patients with liver metastases COCA and found that age was one of the most important variables in predicting the prognosis of liver cancer patients (Kuai et al. 2021). Pei et al. (2020) also found that age impacted the survival of patients with non-metastatic COCA. Herein, although univariate analysis revealed that age in the dataset not stratified by age was not significantly linked to COCA prognosis, we included it in constructing the nomogram. The incidence of early-onset COCA is increasing every year (Tanaka et al. 2023), which has increased by 2% per year since 1994 (Mauri et al. 2019). Compared with late-onset COCA, early-onset COCA, a new subtype of COCA, has unique molecular mechanisms of development, genetic characteristics, and histopathological features (Zaborowski et al. 2021). Most early-onset COCAs are diagnosed in the later stage, it is characterized by poor cell differentiation, and the primary tumor is located in the descending colon. Furthermore, a detailed comparative analysis of gene expression between early-onset COCA and late-onset COCA revealed 88 gene expression changes specific to early-onset COCA (Jandova et al. 2016). In summary, early-onset COCA presents with specific clinical features and has unique molecular development mechanisms. Thus, it was crucial to investigate the unique clinical features and molecular blueprint of early-onset COCA, both of which are important in the clinical diagnosis and treatment of early-onset COCA. The COCA patients in the SEER database were divided into the early-onset COCA and the late-onset COCA groups, and the same factors that impact COCA prognosis were used to construct corresponding nomograms. Based on the area under the ROC, our nomogram accurately predicted COCA prognosis. Notably, the nomogram for the early-onset COCA was more accurate in COCA prognosis prediction. Our findings illustrate differences exist in the factors that impact the prognosis of early-onset and late-onset COCA.

In this study, the relationship between sex and prognosis of COCA patients was not significant. However, demographic variables such as sex and race are one of essential variables in cancer treatment (Goggins and Lo 2012; Shavers and Brown 2002; Zeng et al. 2015). The incidence of COCA is about 20% lower in women than in men (Hultcrantz 2021). Numerous studies have been conducted on the tumor sites of COCA (Siegel et al. 2012; Meguid et al. 2008). The COCA biologies, such as microsatellite instability and differences in gene expression, vary with the cancer site (Papagiorgis et al. 2012; Sun 2021). A study by Ge et al. found significantly different prognoses for COCA at different sites. The OS of patients with right-sided COCA was shorter than those with left-sided COCA (Ge et al. 2019). The precise reason for this difference is not known. However, only a few studies have investigated the specific tumor sites. Specific tumor sites were among the variables incorporated in the constructed nomogram, which increased the accuracy of the nomogram. Nomograms provide a reasonable and reproducible algorithm for individualized prognostic assessment. They have been used for predicting the prognosis of several cancer types, such as pancreatic cancer, gastrointestinal mesenchymal tumor, and gastric cancer, among others (Song et al. 2018a, b; Liu et al. 2016). Combining many clinical variables related to prognosis into the nomogram can reveal comparable staging systems and more disease-specific characteristics. Only significant variables in the univariate and multifactorial analyses were included in constructing the nomograms in the present study. Furthermore, important variables considered in cancer treatment, such as gender, were also included because they are relevant in clinical practice.

The TNM staging system is the standard method for staging COCA (Hari et al. 2013). However, the inherent limitations of the TNM staging system are unavoidable because it only recognizes the T-stage, N-stage, and M-stage when assessing patient prognosis but does not consider other factors that impact patient prognosis (Guevara-Cuellar et al. 2021; Feng et al. 2021). Herein, more variables that impact the OS of COCA patients were included. The ROC curve revealed that the accuracy of the constructed nomogram was higher than that of the conventional TNM staging system.

This study had several limitations. First, given that it was a retrospective study, selection bias in the patient selection process cannot be ruled out. In addition, due to the limited clinical information on patients in the SEER database, more valuable clinical factors, such as specific radiation treatment regimens, surgical access, were not considered in the analysis. Finally, the accuracy of our nomogram was not validated with external data. To override this limitation, our study cohort was divided into the training and validation cohort in a ratio of 7:3, in which the 30% of the population was used for internal validation. The results of the internal validation demonstrated the robustness of the model. Despite these drawbacks, our study has some clear advantages. First, this study included sufficient data, which increased the reliability of our findings. Second, we constructed three nomograms simultaneously, which not only made the results more comparable but also illustrated some differences in the factors that impact prognosis between early-onset COCA and late-onset COCA. Third, the model's accuracy was validated, further demonstrating the stability and reliability of the constructed models. Finally, the dynamic nomograms could predict the OS of COCA patients at all ages.

## Conclusion

Herein, we constructed highly accurate nomograms for predicting the outcomes of early-onset COCA and late-onset COCA. The accuracy of the nomograms was higher than that of the TNM staging system. Among the three nomograms, the nomogram for the early-onset COCA prognosis prediction was the most accurate, followed by the non-age-stratified COCA prognosis prediction nomogram. The nomograms for predicting early-onset and late-onset COCA are useful for clinical management and individualized treatment of patients at different ages.

## Data Availability

The dataset supporting the conclusions of this paper is included in the material of this paper.
